# Novel linkage disequilibrium clustering algorithm identifies new lupus genes on meta-analysis of GWAS datasets

**DOI:** 10.1007/s00251-017-0976-8

**Published:** 2017-02-28

**Authors:** Mohammad Saeed

**Affiliations:** Department of Genomics, Arkana Laboratories, 10810 Executive Center Drive, Suite 100, Little Rock, AR 72211 USA

**Keywords:** Lupus, Linkage disequilibrium, Genome-wide association study, Gene-based tests, Meta-analysis

## Abstract

Systemic lupus erythematosus (SLE) is a complex disorder. Genetic association studies of complex disorders suffer from the following three major issues: phenotypic heterogeneity, false positive (type I error), and false negative (type II error) results. Hence, genes with low to moderate effects are missed in standard analyses, especially after statistical corrections. OASIS is a novel linkage disequilibrium clustering algorithm that can potentially address false positives and negatives in genome-wide association studies (GWAS) of complex disorders such as SLE. OASIS was applied to two SLE dbGAP GWAS datasets (6077 subjects; ∼0.75 million single-nucleotide polymorphisms). OASIS identified three known SLE genes viz. *IFIH1*, *TNIP1*, and *CD44*, not previously reported using these GWAS datasets. In addition, 22 novel loci for SLE were identified and the 5 SLE genes previously reported using these datasets were verified. OASIS methodology was validated using single-variant replication and gene-based analysis with GATES. This led to the verification of 60% of OASIS loci. New SLE genes that OASIS identified and were further verified include *TNFAIP6*, *DNAJB3*, *TTF1*, *GRIN2B*, *MON2*, *LATS2*, *SNX6*, *RBFOX1*, *NCOA3*, and *CHAF1B.* This study presents the OASIS algorithm, software, and the meta-analyses of two publicly available SLE GWAS datasets along with the novel SLE genes. Hence, OASIS is a novel linkage disequilibrium clustering method that can be universally applied to existing GWAS datasets for the identification of new genes.

**Electronic supplementary material:**

The online version of this article (doi:10.1007/s00251-017-0976-8) contains supplementary material, which is available to authorized users.

## Introduction

Complex disorders such as systemic lupus erythematosus (SLE) could be thought of as a mixture of multiple resembling phenotypes, each a result of a separate mutation in genes of a causal pathway (Saeed 2017). Finding a particular gene then depends on the enrichment of a causal mutation carrying haplotype in the study sample. In a genome-wide association study (GWAS), hundreds of thousands of genetic markers are typed creating a multiple testing problem. As a result of random noise, true association signal is hard to decipher. To reduce this error (type I), corrective measures such as the Bonferroni are applied. These may be overly corrective and prevent identification of true associations (type II error), leading to increase in study sample sizes and consequent expense.

GWAS is based on the principle that complex disorders are caused by common variants (frequency >1%) and should therefore be detectable by linkage disequilibrium (LD) mapping using a large number of common variants. Here, this principle is expanded upon by the development of a novel clustering algorithm to identify genes and loci of interest in SLE. As previously shown, gene- or region-based association analysis is an approach that may improve the power of GWAS and allow detection of genes of modest influence (Zhang et al. 2015). It is known that true genetic associations are accompanied by signals from surrounding markers; i.e., single-nucleotide polymorphisms (SNPs) in LD with the susceptibility mutation also have statistically significant *P* values (Martin et al. 2000). Diagrammatically, these surrounding SNPs form a cluster around the causal variant, an “OASIS,” observable in numerous GWAS (Duerr et al. 2006; Edwards et al. 2005; Rioux et al. 2007). Metaphorically, these clusters represent oasis in “gene deserts,” a term usually used to describe absence of coding sequences in DNA; however, in the context of gene, hunting may represent absence of disease susceptibility genes. Hence, this algorithm is termed OASIS.

In this study, OASIS meta-analysis of two SLE GWAS datasets (Harley et al. 2008; Hom et al. 2008) identified three known SLE genes viz. *IFIH1*, *TNIP1*, and *CD44* that were not identified in the original studies. OASIS verified the five genes either of the two studies identified, in both datasets viz. *STAT1*/*STAT4*, *DNASE1L3*/*PXK*, *IRF5*, *BLK*, and *ITGAM*/*ITGAX*. Furthermore, 22 new loci for SLE were identified. Of these, 10 genes were validated by standard single-variant and/or gene-based replication. These new SLE genes include *TNFAIP6*, *DNAJB3*, *TTF1*, *GRIN2B*, *MON2*, *LATS2*, *SNX6*, *RBFOX1*, *NCOA3*, and *CHAF1B.* Hence, OASIS is a novel LD clustering method that can be broadly used to mine existing GWAS datasets for new complex disease genes.

## Methods

### Datasets

GWAS datasets were obtained online from the publicly available dbGAP repository. Meta-analysis of two SLE datasets, phs000202 (Harley et al. 2008) and phs000122 (Hom et al. 2008), was conducted using OASIS. The dataset phs000202 consisted of 706 SLE females and 353 controls and was used for screening (Harley et al. 2008), while phs000122, comprising of 1435 SLE cases and 3583 controls genotyped for 500 K SNPs, was used as the replication dataset (Hom et al. 2008). The *P* values reported in these datasets were based on standard association analysis results as described in the original studies (Harley et al. 2008; Hom et al. 2008).

### OASIS algorithm

In the OASIS algorithm, LD clusters were defined by 200 kb regions. This cutoff has previously been used to define an LD cluster (Bentham et al. 2015). The GWAS file was ordered sequentially by chromosomes and position. The first variant that had a *P* ≤ 0.05 was considered the start of a new OASIS block. SNPs with *P* ≤ 0.05, located within 200 kb of this initial SNP, were counted to form the OASIS score. The 3-sigma (3σ; three standard deviations or a value ≥99.7% of the data) cutoffs were calculated for the −log [P] values and the OASIS scores. This structured the data in two axes (−log [P] and OASIS) and four groups viz. quadrants A–D (Fig. S1). Quadrant A loci were those that crossed the 3σ cutoffs on both axes, quadrant B loci were positive on −log [P] but not on OASIS, quadrant C loci were positive on OASIS scores but not on −log [P], whereas quadrant D loci failed to meet the 3σ cutoffs on either axis.

### OASIS software

The code has been written in Python 2.7.9 (https://github.com/dr-saeed/OASIS/blob/master/OASIS.py) and comprises of three modules. Module 1 reads the GWAS data file and calculates the OASIS scores which are processed by Module 2 to generate the 3σ statistics and graphs (saved in PNG format), for −log [P] values, OASIS scores, and quadrants A–D for each chromosome (Fig. S1). The software can be used for analysis using varying OASIS block sizes, though 200 kb is set as default. GWAS data from dbGAP as well as PLINK output files can be analyzed using the OASIS software. Module 3 is for creating a composite of two GWAS datasets into a single html table with clickable links to NCBI Mapview for easy location of associated regions. Module 3 highlights the overlapping 3σ significant regions in the two datasets with information on their respective quadrants, maximum −log [P] values in those regions, and OASIS scores, besides other valuable data in a tabulated scrollable format. LD has been previously shown to maximally extend to about 2 Mb (Saeed et al. 2009). Therefore, loci overlapping within 2 Mb distance were considered replicated in Module 3 analyses. Moreover, this allowed a reasonable comparison between GWAS datasets, which often use different genotyping platforms.

### OASIS validation using standard analysis

Single-variant replication was performed on SNPs with maximum −log [P] values in loci identified by OASIS. These SNPs in the dataset phs000202 (Harley et al. 2008) were verified for association in the phs000122 (Hom et al. 2008) dataset. Gene-based replication was performed using Gene-based Association Test using Extended Simes procedure (GATES) (Li et al. 2011) as implemented in the KGG software (Li et al. 2010). SNPs were mapped onto genes according to positional information from the NCBI GRCh37 database, and SNPs within 10 kb upstream and 10 kb downstream of each gene were included as well (Zhang et al. 2015). OASIS candidate genes were used as the seed list for GATES verification.

## Results

### Linkage disequilibrium clustering (OASIS)

Data for 258,357 and 489,876 SNPs was available from dbGAP for the datasets phs000202 and phs000122, respectively (Harley et al. 2008; Hom et al. 2008). The input data included information on chromosome number, SNP, location, and *P* value. On OASIS analysis, 5082 regions in the phs000202 dataset were identified, which had at least one variant with a *P* ≤ 0.05. Similarly, 6342 such OASIS blocks were identified in the dataset phs000122. Of these, 292 loci crossed the 3σ cutoffs and were classified in quadrants A, B, or C (159 from the dataset phs000202 and 133 from phs000122). OASIS Module 3 analyses showed that 34 blocks replicated in both datasets. A locus was considered replicated when at least two OASIS blocks from separate datasets were located less than 2 Mb apart. Some of these blocks overlapped, resulting in the identification of a total of 30 SLE loci containing 80 candidate genes (Table S1).

**Fig. 1:**
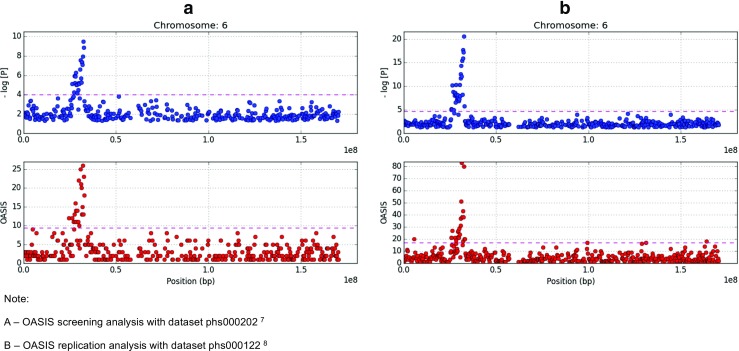
**a**, **b** HLA association with SLE on chromosome 6 shows the high association signal (−log [P] and OASIS score—*y* axes) on chromosome 6 (SNP position in base pairs—*x* axis) for data from both SLE GWAS studies (Harley et al. 2008; Hom et al. 2008). OASIS software automatically generates these graphs as PNG and marks the 3σ cutoff values with a *mauve dashed line* on the *y* axes

**Fig. 2:**
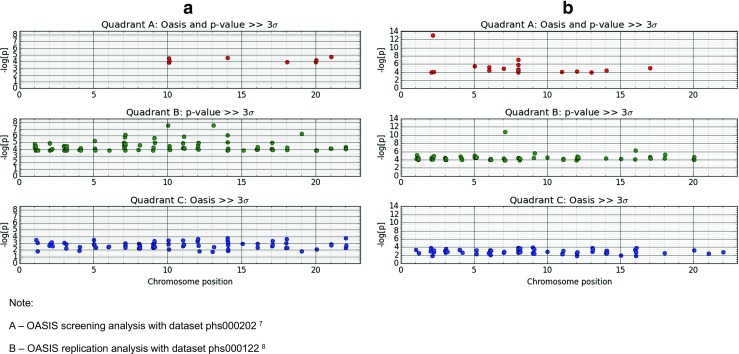
**a**, **b** OASIS genome-wide association in the two SLE datasets shows the −log [P] values for variants across the genome according to their categorization in quadrants (A, B, or C) in the two SLE datasets (Harley et al. 2008; Hom et al. 2008). This is based on the intersection of −log [P] and OASIS score for each variant

The HLA locus on chromosome 6 showed the highest significance in both datasets on OASIS as well as −log [P] analysis (Fig. 1a, b), dwarfing other signals. This affected the 3σ cutoff values, and a second OASIS analysis for non-HLA loci was performed after removing the variants in the HLA locus (25–34 Mb). It is this analysis that resulted in the identification of 292 loci mentioned above (Fig. 2a, b).

In the original studies, five genes/loci were identified using these datasets (Harley et al. 2008; Hom et al. 2008). *STAT1*/*STAT4*, *ITGAM*/*ITGAX* loci, and *IRF5* were found to be associated with SLE in both studies (Harley et al. 2008; Hom et al. 2008). These were verified by OASIS as well. However, *DNAse1L3*/*PXK* locus was originally identified using the dataset phs000202 (Harley et al. 2008) and *BLK* was identified only in the dataset phs000122 (Hom et al. 2008), while OASIS identified these loci in both datasets (Table S1). *BLK* was identified in quadrant B and replicated in quadrants A and C. *DNAse1L3*/*PXK* locus was found to be significant in quadrant B in both screening and replication datasets. This shows that the reason these two loci were missed in one of the studies was due to the use of the stringent Bonferroni correction. Moreover, this finding verifies the application of the 3σ rule to GWAS data.

The success of the OASIS methodology is demonstrated by the identification of known SLE genes not identified using these datasets in the original studies. *IFIH1* screened positive in quadrant C and replicated in quadrant B (locus 2; Table S1). This shows that the OASIS algorithm based on LD clustering is valid, since *IFIH1* shown to be associated using standard analysis in several separate later datasets could not be previously identified in the datasets used here (Gateva et al. 2009; Robinson et al. 2011; Wang et al. 2013). Verification in quadrant B again strengthens the concept of using the 3σ rule. Of even greater significance was the identification, using these GWAS datasets, of the known SLE genes *TNIP1* (locus 7) and *CD44* (locus 15). Both these genes could not be identified in these datasets using standard association analysis, though they have been shown to be associated with SLE in later studies (Gateva et al. 2009; Lessard et al. 2011; Sheng et al. 2015; Yung and Chan 2012; Ramos et al. 2011). However, using the novel OASIS algorithm, these genes were identified and replicated in quadrant C, indicating the sheer usefulness of this analytic technique.

The above findings lend support to the 22 novel SLE loci that were found using OASIS (Table S1). Interesting candidate genes included *TNFAIP6* (locus 1), *DNAJB3* (locus 4), *TTF1* (locus 13), *MON2* (locus 20), *LATS2* (locus 21), *RBFOX1* (locus 26), *NCOA3* (locus 29), and *CHAF1B* (locus 30). Besides their potential pathogenic significance to SLE, the association signals at these loci were mostly concentrated in a narrow region around these genes signifying strong focal LD. The *LATS2* and *CHAF1B* loci are of particular interest because they were either identified or replicated in quadrant A. Most other loci were found in quadrant B or C. Interestingly, *LATS2* was identified in quadrant C and replicated in quadrant A and vice versa for *CHAF1B*. These findings further strengthen the LD-based clustering approach of OASIS.

### Single-variant replication

To validate the loci identified by OASIS in a standard association study model, SNPs that showed the maximum −log [P] values in the OASIS analysis of dataset phs000202 (Harley et al. 2008) were subjected to replication in the phs000122 (Hom et al. 2008) dataset. As shown in Table 1, 7 of 34 SNPs replicated. Of the genes identified in the original studies (Harley et al. 2008; Hom et al. 2008), SNPs in *IRF5*, *ITGAM*/*ITGAX*, and *STAT1*/*STAT4* replicated, while those in the *BLK* and *DNASe1L3*/*PXK* did not. The SNP, rs2785197, with the highest −log [P] value in the OASIS locus for *CD44*, a known SLE gene not identified in the original studies, also replicated and crossed the false discovery rate (FDR). Two novel genes identified using OASIS were nominally significant, *RBFOX1* and *MON2*/*PPM1H*.

**Table 1:** Single-variant replication of OASIS identified loci

SLE gene (OASIS)	SNP ID	*P* value	Rank	FDR	P-FDR
***IRF5***	rs12537284	5.50E−07	1	1.47E−03	1.47E−03
***ITGAM, ITGAX***	rs9888739	6.65E−07	2	2.94E−03	2.94E−03
***IRF5***	rs4728142	1.46E−06	3	4.41E−03	4.41E−03
***STAT1, STAT4***	rs3771327	1.25E−03	4	5.88E−03	4.63E−03
***CD44***	rs2785197	7.13E−03	5	7.35E−03	2.18E−04
*RBFOX1*	rs1881335	3.34E−02	6	8.82E−03	NS
*MIRLET7I, PPM1H, MON2*	rs2704757	4.38E−02	7	1.03E−02	NS

SNPs with the highest −log [*p*] values in the screening dataset (Harley et al. 2008) were verified in the replication dataset (Hom et al. 2008). Seven SNPs replicated at *p* < 0.05 and five SNPs crossed the false discovery rate (FDR). Known SLE genes are in bold

### Gene-based replication

Both datasets (Harley et al. 2008; Hom et al. 2008) were independently subjected to gene-based association using GATES. Of the 80 candidate genes OASIS identified, 24 nominally replicated using GATES at the gene-based level in at least one dataset (Harley et al. 2008; Hom et al. 2008). *IRF5* was the only gene that crossed the Benjamini and Hochberg correction in both datasets (Table 2). Given that known SLE genes from the original studies (Harley et al. 2008; Hom et al. 2008) could not be identified after correction, nominal *P* values were considered evidence of replication. GATES identified known SLE genes *IFIH1* and *TNIP1* in dataset phs000122 (Hom et al. 2008) confirming OASIS findings. However, *CD44* did not replicate using GATES in either dataset in spite of being an established SLE gene. Similarly, *RBFOX1* also did not replicate using GATES. *GRIN2B* and *SNX6* were the two novel OASIS candidate genes that replicated nominally in both datasets (Harley et al. 2008; Hom et al. 2008). *TNFAIP6*, *DNAJB3*, *TTF1*, *MON2*, *LATS2*, *NCOA3*, and *CHAF1B* were verified using GATES in at least one of the datasets (Table 2) (Harley et al. 2008; Hom et al. 2008). Therefore, composite analyses with single-variant replication, gene-based (GATES) and LD clustering (OASIS)-based approaches, have immense potential to mine complex disease genes of low to moderate effect sizes.

**Table 2:** Gene-based association (GATES) of OASIS candidate genes

Chromosome	Position	Gene	P^7^	BH^7^	P^8^	BH^8^
2	152,214,105	*TNFAIP6*			3.59E−02	8.33E−01
2	163,123,588	***IFIH1***			3.40E−04	3.37E−01
2	191,840,262	***STAT1***			2.93E−02	8.10E−01
2	191,894,301	***STAT4***	1.21E−03	4.94E−01	2.42E−12	**5.72E**−**08**
2	234,651,395	*DNAJB3*	7.38E−03	6.74E−01		
3	58,178,352	***DNASE1L3***			4.25E−02	8.51E−01
3	58,318,616	***PXK***	1.32E−02	7.52E−01		
5	150,409,503	***TNIP1***			7.31E−03	6.45E−01
7	31,823,125	*PDE1C*			1.49E−03	4.77E−01
7	128,580,723	***IRF5***	2.92E−06	**1.81E**−**02**	8.25E−11	**9.75E**−**07**
8	11,351,520	***BLK***	9.99E−02	9.04E−01	2.57E−06	**1.22E**−**02**
9	135,250,936	*TTF1*	1.10E−04	1.70E−01		
12	12,268,960	*LRP6*			9.80E−04	4.51E−01
12	13,714,409	*GRIN2B*	4.48E−02	8.47E−01	4.65E−02	8.59E−01
12	31,079,837	*TSPAN11*	4.80E−04	4.71E−01		
12	62,860,596	*MON2*	1.33E−02	7.52E−01		
13	21,547,175	*LATS2*			1.93E−03	5.36E−01
14	35,030,617	*SNX6*	4.43E−02	8.47E−01	4.93E−02	8.68E−01
14	73,436,152	*ZFYVE1*	1.39E−02	7.59E−01		
16	31,271,287	***ITGAM***	7.00E−04	4.74E−01	5.98E−06	**2.02E**−**02**
16	31,366,454	***ITGAX***	3.35E−02	8.29E−01	4.81E−06	**1.90E**−**02**
20	46,130,600	*NCOA3*	1.80E−03	5.64E−01		
20	46,286,149	*SULF2*	6.55E−03	6.73E−01		
21	37,757,688	*CHAF1B*			7.02E−03	6.42E−01

GATES was used to carry out gene-based association of the 80 OASIS candidate genes identified in 30 loci. Known SLE genes and the *p* values that crossed the Benjamini and Hochberg (BH) correction are highlighted in bold. Nominally significant *p* values for the genes in the screening (Harley et al. 2008) and replication (Hom et al. 2008) SLE GWAS datasets are shown

## Discussion

In this study, meta-analysis of two dbGAP GWAS datasets (Harley et al. 2008; Hom et al. 2008) using OASIS identified three known SLE genes viz. *IFIH1*, *TNIP1*, and *CD44* that could not be discovered previously using these datasets. The algorithm verified the five genes either of the two SLE studies identified, in both datasets viz. *STAT1*/*STAT4*, *DNASE1L3*/*PXK*, *IRF5*, *BLK*, and *ITGAM*/*ITGAX*. Furthermore, 10 new SLE genes were identified and validated using single-variant and gene-based analyses. Hence, OASIS is a unique method of GWAS meta-analysis that can be employed to identify new genes and loci.

Complex disorders such as the SLE are diverse, manifesting more like syndromes than singular diseases (Saeed 2017). Therefore, GWAS cohorts in effect, pool multiple mutations in separate genes of a mixture of resembling phenotypes. For instance, *DNAse1L3* mutations code for both SLE and hypocomplementemic urticarial vasculitis (HUVS), phenotypes that are clinically classified separately but nonetheless substantially overlap (Al-Mayouf et al. 2011; Ozçakar et al. 2013). When assumed this way, genome-wide corrections such as Bonferroni lose power to identify the myriad of variants responsible for the phenotypic heterogeneity of a complex disorder. Similarly, more than one gene may exist at a locus for a complex disorder as exemplified by the identification of *ANXA6* as a SLE gene located immediately downstream of *TNIP1* (Zhang et al. 2015). Hence, LD-based clustering algorithms such as OASIS that focus association signals to loci are of critical importance and can be followed up by biological studies for verification of particular genes.

Despite the identification of large number of genes in multiple comprehensive SLE GWAS and candidate gene studies, they together explain only 15% of SLE heritability (Bentham et al. 2015). One possible reason for this discrepancy could be increased numbers of false negatives in GWAS due to stringent corrections such as Bonferroni. The 3σ rule is a time-tested statistic that, as shown here, can potentially overcome the problem of false negatives in GWAS. OASIS identified *BLK* and *DNAse1L3*/*PXK* loci using the 3σ statistic in both SLE datasets, though these had been missed previously in one of the datasets (Harley et al. 2008; Hom et al. 2008). Thus, the 3σ statistic applied to GWAS datasets without any correction provides greater opportunity to identify novel genes. Hence, no corrections were applied to the results even though OASIS incorporates two different mechanisms to observe the same underlying phenomenon, i.e., LD (Streiner and Norman 2011). Possibly as this new method of gene discovery evolves, it will become clearer as to what types of correction methods may be applied. However, it has been previously argued that corrections should not be made for multiple comparisons in order to reduce the type II error (Rothman 1990). This was aptly demonstrated in the gene-based replication of OASIS loci using GATES. The only gene that survived the Benjamini and Hochberg correction in both datasets was *IRF5*, whereas several genes were significant at the uncorrected nominal level (Table 2).

Standard association analysis is based on the *χ* ^2^ statistic, which is skewed by low-frequency alleles and can result in highly significant *P* values (type I error). Clustering of significant SNPs, as in OASIS, reduces the possibility of false positive associations (type I error). OASIS is an algorithm that functions in a manner akin to global tests of association such as gene- or pathway-based tests (Christoforou et al. 2012; Neale 2004). The identification in quadrant C, of SLE genes *TNIP1* and *CD44*, validates the novel strategy of LD clustering in OASIS.

Replication has been the classic mechanism of verification to avoid type I errors. Here, the OASIS findings were replicated using standard single-variant and gene-based analyses. The verification of known SLE genes (*IFIH1*, *CD44*, and *TNIP1*/*ANXA6*) in datasets that did not previously identify them is categorical evidence of the validity of OASIS as a novel gene-hunting tool. About 20% of variants in OASIS-identified loci replicated in single-variant analysis, whereas 30% of OASIS candidate genes (24 of 80) replicated using GATES. This together led to the validation of 60% (18 of 30) of the OASIS-identified loci.

These validated SLE genes are biologically relevant. *TNFAIP6* codes for the protein TSG-6 which inhibits presentation of chemokines on endothelial surfaces leading to decreased infiltration of T cells and dendritic cells during inflammation (Dyer et al. 2016). Loss-of-function variants in *TNFAIP6* may predispose to SLE. TTF1 is a nucleolar factor that controls transcription of the ribosomal RNA genes. This process determines the cell-cycle state from proliferation to apoptosis (Lessard et al. 2012). TTF1 levels are regulated by MDM2-mediated ubiquitinylation (Lessard et al. 2012). MDM2 is known to promote SLE in a murine model (Allam et al. 2011). Possibly, *TTF1* variants that decrease ubiquitinylation by MDM2 may lead to lymphoproliferation in a manner similar to MDM2 overexpression, leading to SLE (Allam et al. 2011). LATS2 promotes apoptosis by shunting p53 to pro-apoptotic promoters (Aylon et al. 2010). LATS2 is also known to function as a negative regulator of TNF-α-induced NF-κB activation (Yao et al. 2015). *CHAF1B* is involved in epigenetic control of chromatin dynamics during cell cycling, and its inhibition leads to apoptosis and accumulation of double-strand breaks (Nabatiyan and Krude 2004). MON2 and SNX6 are involved in endosome-to-Golgi trafficking (Mahajan et al. 2013). SNX6 traffics members of the TGF-β family of receptor serine-threonine kinases (Parks et al. 2001). *NCOA3* codes for the SRC-3 protein that plays an important role in maintenance of T cell function (Li et al. 2012).

The OASIS algorithm provides an alternative to increasing sample sizes for GWAS to ascertain variants with low to moderate effect sizes. This is made possible by composite analysis based on two axes (−log [P] and OASIS blocks) divided into association quadrants. This unifies two aspects of the LD phenomenon—strength of association of a single-variant and the number of significant genetic markers (Fig. S1). Caveats with the algorithm are that the results may be affected by population-based LD, leading to high OASIS scores in regions such as the HLA genomic segments (Fig. 1). Moreover, the number of SNPs genotyped at a locus may skew the scores as well. Population stratification in case control studies will likely affect OASIS results as it does standard association analysis.

In summary, OASIS is a novel LD clustering algorithm described here that can be widely applied for mining existing GWAS datasets to identify candidate genes in an efficient, low-cost way. These candidates can be subsequently replicated in other studies such as single-variant, gene-based analyses and biological studies. Here, OASIS was applied to two dbGAP SLE GWAS datasets and identified 8 known and 10 novel SLE genes. OASIS was verified using two sets of analysis viz. single-variant replication and gene-based analyses using GATES. This study also underscores the need to make more GWAS datasets publically available for further development of novel analytic tools. Taken together, these findings highlight the novelty and efficiency of LD-based clustering approaches, such as the OASIS algorithm, for GWAS meta-analysis of complex disorders.

## Electronic supplementary material

Table S1. OASIS Associated SLE loci replicated in two GWAS datasets Shows the 30 replicated SLE genes/loci in the two studies (Harley et al. 2008; Hom et al. 2008). These loci containing OASIS blocks within 2 Mb of each other are separated by bold horizontal lines. Data regarding the chromosomal location and size of blocks and the strength of the association signal (−log [P], OASIS scores and Quadrants) is shown. The candidate genes are based on the location and distribution of the association signal (SNPs) on NCBI Mapview. Known SLE genes are highlighted in black and bold and new SLE genes in blue and bold. (XLSX 16 kb)

Figure S1. OASIS Quadrants Describes the OASIS Quadrants A, B and C diagrammatically. The scatter graph shows −log [P] values plotted against OASIS scores. (PPTX 76 kb)
