# Supplementary material for: Novel linkage disequilibrium clustering algorithm identifies new lupus genes on meta-analysis of GWAS datasets
Source: Immunogenetics. 2017 Feb 28;69(5):295–302. doi: 10.1007/s00251-017-0976-8 (PMC5400794; doi:10.1007/s00251-017-0976-8)
Supplement: Supplementary file 2 — Describes the OASIS Quadrants A, B and C diagrammatically. The scatter graph shows −log [P] values plotted against OASIS scores. (PPTX 76 kb) [file 251_2017_976_MOESM2_ESM.pptx]

## Slide 1
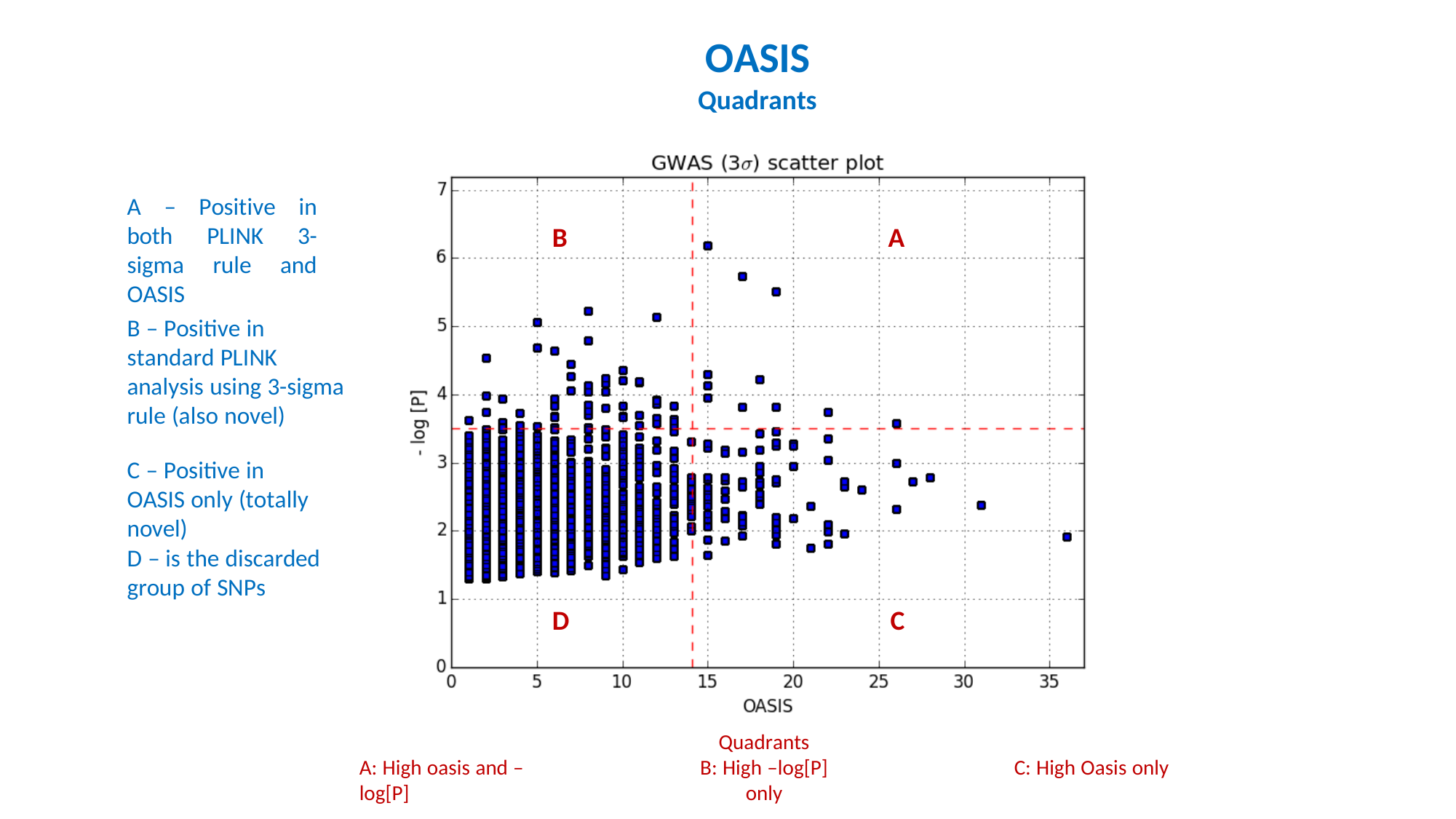

OASIS
Quadrants
A – Positive in both PLINK 3-sigma rule and OASIS
B – Positive in standard PLINK analysis using 3-sigma rule (also novel)
C – Positive in OASIS only (totally novel)
D – is the discarded
group of SNPs
A
B
D
C
Quadrants
B: High –log[P] only
A: High oasis and –log[P]
C: High Oasis only
